# Performance characteristics of an automated, high-throughput RT-PCR assay for the detection of *Candida auris* on 3-point and nasal swabs

**DOI:** 10.1128/spectrum.02114-24

**Published:** 2025-02-14

**Authors:** Nhi T. Nhan, Tianxi Liu, Abdulaala A. Almushajrah, Ashish Mozumder, Momka Narlieva, Wendy A. Szymczak, Phyu M. Thwe, Erika P. Orner, Doctor Y. Goldstein

**Affiliations:** 1 Department of Pathology, Montefiore Medical Center, Bronx, New York, USA; 2 Department of Pathology, Albert Einstein Medical School, Montefiore Medical Center, Bronx, New York, USA; MultiCare Health System, Tacoma, Washington, USA

**Keywords:** *Candida auris*, PCR, screening, diagnostic

## Abstract

*Candida auris* is a multidrug-resistant yeast responsible for invasive infections with high mortality rates, primarily spread through prolonged colonization on biotic and abiotic surfaces and traveling. Effective control necessitates comprehensive screening protocols, as recommended by the Centers for Disease Control and Prevention, which endorses a real-time polymerase chain reaction-based assay for *C. auris* screening. This study evaluates the performance of this assay on the Hologic Panther Fusion System using nasal and 3-point swab specimens (nares/axilla/groin) compared with culture. The assay was assessed for accuracy, sensitivity, specificity, and reproducibility, alongside an evaluation of probe primer reagent (PPR) onboard stability over 30 working days. Analytical sensitivity studies determined limits of detection of 1.95 Log CFU/mL for nasal swabs and 2.18 Log CFU/mL for 3-point swabs, both with >95.0% confidence intervals. The assay demonstrated 100% specificity (*n* = 25), with no false positives from genetically similar or clinically relevant species, and no significant interference from co-infecting microbes on cycle threshold (CT) values. Both swab types exhibited high intra- and inter-reproducibility, with low coefficients of variation (1.79% and 1.06%, respectively). The assay detected *C. auris* in 100% of 108 spiked-positive samples across both swab types. Clinical analysis showed 100% concordance with culture for 3-point swabs. Additionally, the nasal swab method showed 96.0% overall agreement, with 86.2% sensitivity and 100.0% specificity. The PPR mixes remained stable over 30 working days, with no significant CT value changes. This study confirms the assay’s robust sensitivity, specificity, reproducibility, and accuracy for both nasal and 3-point screening swabs.

**IMPORTANCE:**

*Candida auris* is a multidrug-resistant yeast responsible for severe infections with high mortality rates. Rapid and accurate detection is critical for preventing the spread of this pathogen in healthcare settings. This study assesses the performance of an automated real-time PCR screening assay for detecting *C. auri*s using nasal and 3-point swabs. The findings demonstrate the assay's high sensitivity, specificity, and reproducibility, making it a valuable tool for infection control. By providing a reliable and efficient screening method, this assay can significantly enhance efforts to control *C. auris* outbreaks, ultimately improving patient outcomes and reducing the spread of this dangerous pathogen.

## INTRODUCTION

*Candida auris* has emerged as a multidrug-resistant fungal organism of high concern due to its ability to cause invasive infections with high resistance and mortality rates along with its potential to cause outbreaks in healthcare settings. *C. auris* is the first pathogenetic yeast that was listed as an urgent threat by the U.S. Centers for Disease Control and Prevention (1). Following the COVID-19 pandemic, hospitals around the world saw a significant increase in *C. auris* infections, affecting both critically ill patients and those recovering in post-acute care facilities (2–7). Five percent to 25% of patients with *C. auris* colonization can develop life-threatening bloodstream infections (8–10). *C. auris* can persist on environmental surfaces for prolonged periods of time due to its capacity to form adherent biofilms, which have nutritional advantages and penetration protection against different concentrations of disinfectants and antifungals (11, 12). It is important to reinforce effective infection control and identification of patients to limit the risk of transmission within healthcare facilities and reduce the incidence of healthcare-associated outbreaks.

Traditional culture-based methods are time-consuming, often taking days for conclusive results, which can lead to delays in implementing appropriate infection control measures. Real-time polymerase chain reaction (RT-PCR)-based rapid screening, which targets the *C. auris* internal transcribed spacer region 2 (ITS2) gene, has shown better performance characteristics and workflow efficiency with faster turnaround time compared with traditional culture (13). The Centers for Disease Control (CDC) has published a real-time PCR-based assay for the detection of *C. auris,* and some have adapted its use to high-throughput, automated PCR systems such as the Hologic Panther Fusionx (13, 14). Here, we evaluate the analytical performance characteristics of this real-time PCR-based screening assay on both nasal alone and 3-point collection (nasal, axilla, and groin) screening swabs by assessing accuracy, analytical sensitivity (the limit of detection [LoD]), and specificity, reproducibility, and reagent on-board stability.

## MATERIALS AND METHODS

### Specimen collection

Two specimen types were utilized in this study. Both nasal swab specimens and 3-point (nasal, axilla, and groin) swab specimens were collected and stored in 1 mL Liquid Amies Elution Swab (ESwab) Collection/Transport System tubes (BD: Sparks, MD) at room temperature. Nasal-alone specimens were collected from the anterior nares by swabbing both nares with the same swab before placing the swab into the ESwab tube. 3-point swab specimens were collected by first swabbing both anterior nares, then bilaterally swabbing both axillae, and lastly bilaterally swabbing the groin, all using the same swab before placing the swab into the ESwab tube.

### *C. auris* culture

A 10 µL aliquot of the clinical specimen, collected in Liquid Amies Elution Swab (ESwab), was inoculated onto CHROMagar Candida Plus (CHROMagar: Paris, France) using a sterile calibrated loop and struck for isolated colonies. The inoculated plate was incubated aerobically at 30°C–37°C for 24–48 h. Presumptive identification of *C. auris* was based on the characteristic appearance of light blue colonies with a distinct blue halo observed from the plate’s back side. All potential *C. auris* isolates were subjected to confirmatory identification using matrix-assisted laser desorption/ionization time-of-flight mass spectrometry (MALDI-TOF MS). Briefly, a single colony of presumptive *C. auris* was spotted on a disposable MBT Biotarget 96 plate (Bruker: Billerica, MA) using a sterile, wooden toothpick. One microliter of formic acid (Sigma-Aldrich: St. Louis, MO) was then pipette on top of the spotted colony and allowed to dry in ambient air. Once dry, 1 µL of α-Cyano-4-hydroxycinnamic acid (HCCA) matrix (Bruker: Billerica, MA) was overlayed on top of each spot and allowed to dry. The Biotarget plate was then run on the MALDI Biotyper (Bruker: Billerica, MA) and analyzed using the FDA-approved MALDI Biotyper CA library. Only identifications with scores of 2.0 or higher were considered acceptable and accurate.

### *C. auris* real-time PCR screening assay

The C. *auris* real-time PCR assay was developed and published by the CDC to detect the *C. auris* internal transcribed spacer region 2 (ITS2) within its ribosomal gene (15). We modified this protocol to run on the Hologic Panther Fusion System using Open Access software. Briefly, we incorporated the CDC-designed *C. auris* primers and probes with FAM fluorescence dye and utilized the Hologic DNA internal control (IC) primers and probes (Cat No. PRD-04306 and PRD-04308), which are detected in the Quasar 705 channel. The *C. auris* primers and probe are as follows: 5′-CAG ACG TGA ATC ATC GAA TCT-3′, 5’- TTT CGT GCA AGC TGT AAT TT-3′, and 5’-/56-carboxyfluorescein (FAM)/AAT CTT CGC /ZEN/GGTGGCGTTGCA TTC A /3IABkFQ/−3’ (15). Thermocycling conditions were modified as follows: 1 cycle of 95^◦^C for 18 s, 45 cycles of 95^◦^C for 5 s, and 60^◦^C for 30 s. Each primer probe reagent (PPR) mixture contained 50 mM KCl, 4.0 mM MgCl_2_, 10.0 mM Tris, 0.6 µM concentration of each *C. auris* primer and Hologic DNA IC primer, and 0.4 µM concentration of each *C. auris* probe and Hologic DNA IC probe.

Following the routine Open Access protocol on the Panther Fusion system, 500 µL of clinical sample was added into 710 µL of Aptima Hologic Lysis Tubes (Cat No. PRD-06952) before loading the tube onto the instrument. Panther Fusion Extraction Reagents-X (Cat No. PRD-04477) was selected for nucleic acid extraction, given its demonstrated performance on DNA-only applications. The Hologic IC-X (Cat No. PRD-04308) was added into Panther Fusion Capture Reagent-X by the instrument. This method utilizes oligonucleotide-conjugated magnetic beads for targeted capture of specific DNA sequences from a crude sample. Following hybridization, a magnetic field facilitates the separation of the captured DNA from the sample matrix. Subsequent washing steps eliminate non-specific interactions, whereas a final elution step yields purified DNA. The nucleic acid is subsequently eluted into 50 µL, and 5 µL is amplified via the real-time PCR protocol described above.

### Analytical performance assessment

The *C. auris* isolate #0389 from the CDC AR Bank was used to assess the analytical sensitivity of the real-time PCR assay. *C. auris* was grown at 37°C for 48 h on a Sabouraud Dextrose agar (SDA) plate (BD: Sparks, MD). Multiple colonies were diluted in a 3.0 mL BD Phoenix ID saline tube (BD: Sparks, MD) to a 1.0 McFarland (suspension was measured using BD PhoenixSpec nephelometer). The starting concentration of the 1.0 McFarland standard was calculated to be 2.0 × 10^7^ CFU/mL by averaging colony counts obtained from triplicate plates of five 10-fold dilutions of the standard.

#### Linearity

Analytical linearity of the 3-point and the nasal-alone ESwab specimens was assessed for the real-time PCR-based screening assay on the Hologic Panther Fusion System. A 1.0 McFarland of spiked *C. auris* specimen (CDC AR-Bank #0389) was serially diluted 10-fold using both negative 3-point and negative nasal-alone specimen pools. Six concentrations were tested ranging from 1.92 to 6.92 Log CFU/mL (6.92, 5.92, 4.92, 3.92, 2.92, and 1.92 Log CFU/mL). Three separate extractions of each collection type were done, and each was tested in triplicates for each concentration level across 3 consecutive days.

#### Limit of detection

Probit analysis was employed to determine the LoD for *C. auris* in both nasal swab (nasal-alone) and 3-point Eswab specimens using MedCalc software (MedCalc Software Ltd, Ostend, Belgium; https://www.medcalc.org; 2020). The study involved testing a spiked *C. auris* specimen diluted across multiple concentrations: 1.18, 1.48, 1.65, 1.88, and 2.13 Log CFU/mL. Each concentration level was tested in eight replicates for both collection types. Probit analysis was subsequently applied to determine concentrations yielding more than 95.0% confidence intervals (CI) for detection probability. Concentrations identified through probit analysis (>95.0% CI) were further validated by testing twenty replicates for each collection type.

#### Reproducibility

Analytical reproducibility was evaluated by testing available clinical patient specimens along with one negative specimen from each collection type in triplicate within the same day and on 3 consecutive days. Testing was conducted using different Hologic Panther Fusion instruments and performed by different technologists to assess reproducibility across various conditions.

#### Specificity

Analytical specificity was assessed using genetically related organisms and clinically relevant organisms that are typically present in each specimen matrix. A total of 25 control strains and clinical strains were utilized during this study (Table 2). All strains were stored at −80°C. Bacterial strains were grown on commercially prepared blood agar plates (BD: Sparks, MD), and yeast strains were grown on commercially prepared SDA plates. All plates were incubated at 37°C for 24–48 h. Standardized concentrations of all strains were produced by inoculating BD Phoenix ID saline tubes with enough colonies to produce a 0.5 McFarland (±0.05) as measured by a BD PhoenixSpec nephelometer (BD: Sparks, MD). These prepared organism-containing solutions were diluted 1:10 with either a negative 3-point Eswab matrix or a negative nasal Eswab matrix to simulate the test matrix encountered during clinical use. A review of historically positive specimens for *C. auris* tested by PCR allowed for an estimation that clinical isolates typically presented in the range of 5.00 Log CFU/mL. Based on this observation, 5.00 Log CFU/mL of *C. auris* was spiked into both ESwab matrices along with each matrix also spiked with non-*C*. *auris* species to further evaluate potential interference from co-colonizing organisms and the impact on PCR reactions.

#### Accuracy

Analytical accuracy was evaluated in two ways. First, the clinical accuracy of the PCR assay was done by comparing PCR results against the established culture method for 3-point collection swabs. One hundred and twenty-seven clinical Eswab specimens were collected from 127 patients using the standard 3-point collection method recommended by New York State (NYS) for *C. auris* screening (nasal, axila, and groin sampling). Of these, 45 were culture positive and 82 were culture negative.

Second, the accuracy of nasal alone swab specimens was compared with 3-point swab collection. Ninety-five remnant nasal Eswab specimens originally collected for methicillin-resistant *Staphylococcus aureus* (MRSA) screening were collected from 95 patients who also previously tested for *C. auris* colonization using the standard 3-point ESwab method. Due to limitations of ESwab volume, the remnant nasal-alone swab samples could not be cultured for confirmation. Therefore, the evaluation of accuracy was based on the previously obtained results from the 3-point collection method.

#### PPR on-board stability

A comprehensive stability study was conducted to evaluate the on-board stability of the PPR mixes at ambient temperature on the Hologic Panther Fusion instrument. This study involved testing *C. auris* samples with a concentration of 5.0 Log CFU/mL stored at −20°C so that any observed variability could not be attributed to sample factors. Over a period of 30 testing days, the PPR mixes were maintained on the instrument, and the CT values were monitored and analyzed to detect any fluctuations. This monitoring aimed to assess the stability and performance consistency of the PPR mixes specifically under the ambient conditions of the operating instrument.

#### Data analysis

All sensitivity, probit analysis, specificity, positive percent agreement, negative percent agreement, and confidence interval (CI) were analyzed using GraphPad Prism version 10.0.0 for Windows (GraphPad Software, Boston, Massachusetts USA, www.graphpad.com) and MedCalc Statistical Software version 22.030 (MedCalc Software Ltd, Ostend, Belgium; https://www.medcalc.org; 2020). The software was accessed on 15th May 2024.

## RESULTS

### Linearity and LoD

**Fig 1:**
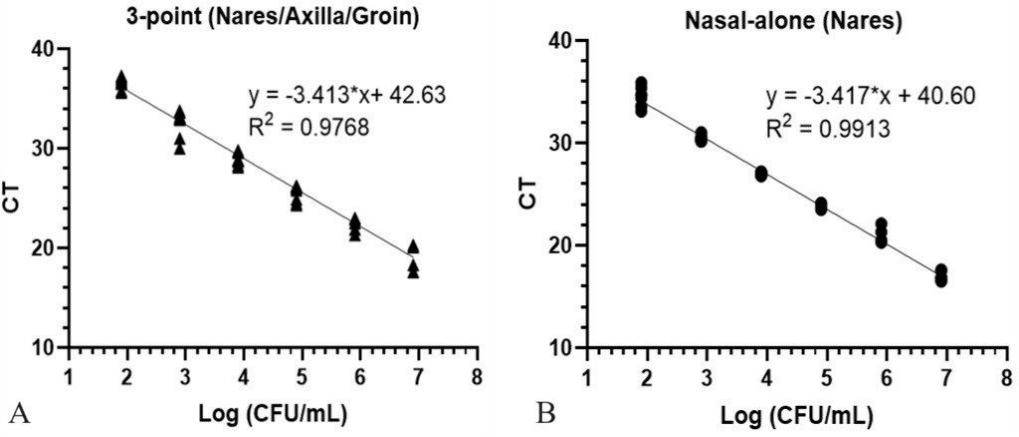
Linearity was established across the range of *C. auris* concentrations from 2.22 to 7.22 Lo g CFU/mL in (A) 3-point ESwab and (B) nasal-alone ESwab.

Analytical linearity demonstrated a high correlation coefficient (R^2^) in both nasal-alone and 3-point ESwab specimens, 0.99 and 0.98, respectively (Fig. 1). The linear regression model of both collection types exhibited high amplification efficiency for both 3-point and Nares at 96.0%. The y-intercept of the nasal-alone linear regression model had lower cycle threshold (CT) compared with 3-point linear model, which suggests a lower theoretical limit of detection (LoD) for this assay in nares matrix.

**Fig 2:**
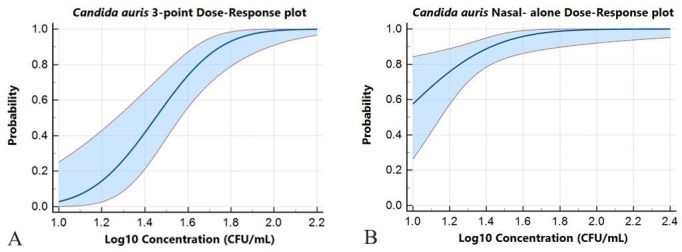
Probit analysis at multiple levels of low concentration with the lowest at 1.18 Log CFU/mL. (**A**) 3-point Eswab: Probit analysis determined the LoD to be 1.88 Log CFU/mL with a mean CT value of 36.77 ± 1.32. (**B**) Nasal-alone Eswab: Probit analysis determined the LoD to be 1.65 Log CFU/mL with a mean CT value of 35.20 ± 1.50.

The probit analysis was conducted in both nasal-alone and 3-point collection types at multiple levels of low concentration with the lowest at 1.18 Log CFU/mL (Fig. 2). Following this, concentrations demonstrating >95.0% CI were identified for both collection types. Subsequent confirmation involved twenty replicates for each collection type, validating LoD concentrations of 1.65 Log CFU/mL for nasal-alone swab specimens and 1.88 Log CFU/mL for 3-point ESwab specimens. The LoD of 3-point and nasal-alone Eswab displayed a mean CT value of 36.77 ± 1.32 and 35.20 ± 1.50, respectively.

### Intra- and inter-reproducibility

The reproducibility of PCR CT values was assessed using various Hologic Panther Fusion instruments and different technologists, demonstrating 100% reproducibility across all tested variables. The average coefficient of variance (CV) of the PCR CT values was 1.79% in 3-point (mean ± SD, 21.3 ± 2.0) and 1.06% in nares matrix, (mean ± SD, 24.3 ± 1.1), which indicated a very tight performance and limited variability across runs (Table 1). All results from the 3-point ESwab specimens and nasal-alone ESwab specimens were highly reproducible with consistent CT values across same-day and all 3 days of testing. The observed standard deviations (SD) indicate that the 3-point specimens (± 2.0) exhibited a broader range of variability in CT values, reflecting more fluctuation in results, whereas the nasal-alone specimens (± 1.1) demonstrated a narrower range of variability and greater consistency. The negative clinical specimens from both collection methods remained consistently undetected across both intra- and inter-day testing.

**TABLE 1:** Intra- and Inter-assay reproducibility of 3-point and nasal alone ESwab collection types

Collection type	Sample ID	Observed mean CT	Within-day		Between-day		Total	
			SD	%CV	SD	%CV	SD	%CV
Nares/Axilla/Groin	Clinical sample 1	19.3	0.12	0.55	0.61	3.19	0.37	1.87
	Clinical sample 2	23.3	0.15	0.66	0.66	2.82	0.41	1.71
Nasal	Clinical sample 3	24.8	0.06	0.23	0.20	0.81	0.13	0.52
	Clinical sample 4	23.7	0.00	0.00	0.76	3.18	0.38	1.59

### Specificity

The *C. auris* PCR demonstrated 100% specificity without any documented off target positivity across 25 tested strains, including control and clinical isolates of diverse pathogenic microorganisms (Table 2). Furthermore, the positivity for *C. auris* was not impacted when other microbes besides *C. auris* were present, as there was no significant difference in CT values (SD = ±0.20 for 3-point and ±0.10 for nasal-alone) between *C. auris* alone and *C. auris* with other organisms (Table S1 in the supplemental material). There was no interference from other related organisms that might affect real-time PCR reactions for both matrices.

**TABLE 2:** Specificity of 3-point and nasal-alone ESwab with other organisms

Organism	Source	LDT*—C. auris* PCR Result	
		Nares/Axilla/Groin	Nasal-alone
*Candida duobushaemulonii*	CDC AR Bank # 0391	Not detected	Not detected
	CDC AR Bank # 0392	Not detected	Not detected
*Candida haemulonii*	CDC AR Bank # 0393	Not detected	Not detected
	CDC AR Bank # 0395	Not detected	Not detected
*Kodameae ohmeri*	CDC AR Bank # 0396	Not detected	Not detected
*Pichia kudriavzevii (Candida krusei*)	CDC AR Bank # 0397	Not detected	Not detected
	Clinical specimen	Not detected	Not detected
*Clavispora (Candida) lusitaniae*	CDC AR Bank # 0398	Not detected	Not detected
	Clinical specimen	Not detected	Not detected
*Candida albicans*	ATCC 14053	Not detected	Not detected
	Clinical specimen	Not detected	Not detected
*Candida tropicalis*	ATCC 950	Not detected	Not detected
	Clinical specimen	Not detected	Not detected
*Candida parapsilosis*	ATCC 22019	Not detected	Not detected
	Clinical specimen	Not detected	Not detected
Methicillin-resistant *Staphylococcus aureus* (*MRSA*)	ATCC 43300	Not detected	Not detected
	Clinical specimen	Not detected	Not detected
Methicillin-susceptible *Staphylococcus aureus (MSSA*)	ATCC 29213	Not detected	Not detected
	Clinical specimen	Not detected	Not detected
*Staphylococcus epidermidis*	ATCC 12228	Not detected	Not detected
	Clinical specimen	Not detected	Not detected
*Staphylococcus hominis*	Clinical specimen	Not detected	Not detected
	Clinical specimen	Not detected	Not detected
*Escherichia coli*	ATCC 13846	Not detected	Not detected
	Clinical specimen	Not detected	Not detected

### Accuracy

#### Culture and 3-point PCR accuracy comparison

The results demonstrated a 100% agreement between the culture method and the PCR assay for the 127 3-point collection type where the positive predictive value (PPV) and negative predictive value (NPV) were both 100%, underscoring its efficacy in accurately identifying *C. auris* in clinical specimens (Table 3).

**TABLE 3:** *Candida auris* culture and PCR results for 3-point ESwab samples

	Culture positive (*C. auris*)	Culture negative (*C. auris*)	Total
PCR positive (*C. auris*)	45	0	45
PCR negative (*C. auris*)	0	82	82
Total	45	82	127

#### 3-Point PCR and nasal PCR accuracy comparison

Nasal specimens originating from patients previously diagnosed with *C. auris* colonization using the routine 3-point ESwab collection and having alternate testing on nasal swabs were evaluated. The mean CT value for traditional 3-point clinical specimens was 26.5 (range: 17.4–38.1), whereas nasal specimens exhibited a mean CT of 23.0 (range: 17.7–36.2). *C. auris* was detected in 25 of the 29 presumptive positive specimens (86.3%) nasal-alone specimens and was not detected in 66/66 (100.0%) presumptive negative nasal-alone specimens (Table 4). Among patients diagnosed with *C. auris* using the traditional 3-point collection method, nasal swabs demonstrated a 100% PPV and a 94.3% NPV. Overall, the agreement between the nasal swab method and the 3-point collection method was 96.0%.

**TABLE 4:** *Candida auris* PCR results in 3-point ESwab and nasal ESwab samples

	3-Point PCR positive (*C. auris*)	3-Point PCR negative (*C. auris*)	Total
Nasal PCR positive (*C. auris*)	25	0	25
Nasal PCR negative (*C. auris*)	4	66	70
Total	29	66	95

### PPR on-board stability

**Fig 3:**
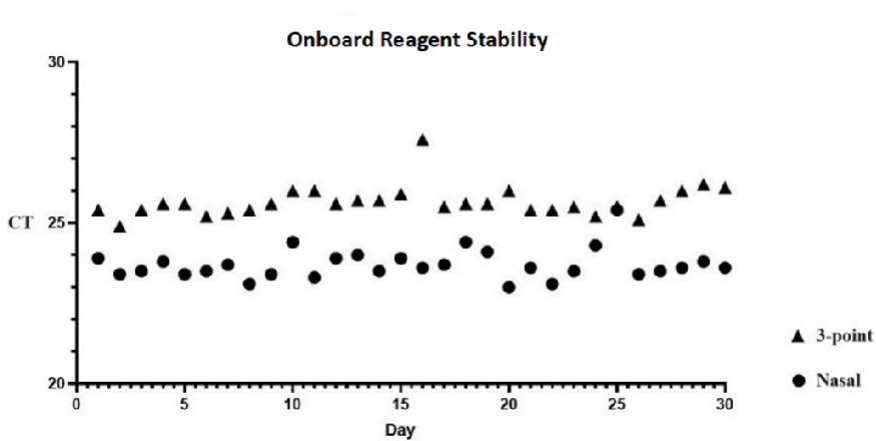
Stability of onboard PPR reagents. CT values of the same *C. auris* 3-point and nasal-alone (nares) specimens tested by onboard PPR reagents left at ambient temperature over the span of 30 days (Mean ± SD = 25.7 ± 0.5 for 3-point, 23.7 ± 0.5 for nasal).

The stability results showed no significant CT fluctuations between the days. The mean CT value for the 3-point ESwab specimens was 25.7 with an SD of ±0.5, whereas the nasal-alone ESwab specimens had a mean CT value of 23.7 with an SD of ±0.5 over the 30-working day period (Fig. 3).

## DISCUSSION

Automated, high-throughput methods for the detection of *C. auris* colonization are essential to epidemiological control efforts, particularly given the pathogen’s high mortality rates and ability to cause widespread outbreaks in healthcare settings. Our study evaluated the performance characteristics of a real-time PCR-based assay for *C. auris* detection on the Hologic Panther Fusion System, for both nasal-alone Eswab and 3-point (nares/axilla/groin) Eswab. The findings underscore the importance of accurate, sensitive, and reproducible screening methods in controlling the spread of this multidrug-resistant yeast.

The analytical sensitivity study revealed that the LoD for the nasal swabs was 1.65 log CFU/mL and 1.88 log CFU/mL for the 3-point swabs. Both collection methods demonstrated high specificity, with no false positives or interference from genetically similar or clinically relevant species. The specificity was 100%, indicating that the assay is robust against cross-reactivity with other microorganisms. Reproducibility assessments showed consistent PCR CT values across different instruments, technologists, and testing days. The CV for intra- and inter-assay reproducibility was low, with values of 1.79% for 3-point swabs and 1.06% for nasal swabs. This high reproducibility is crucial for ensuring reliable detection of *C. auris* in clinical settings, where consistent results are necessary for effective infection control measures.

The accuracy of detecting *C. auris* using different specimen collection methods was thoroughly assessed in this study, focusing on both the 3-point ESwab and nasal-alone ESwab methods. The PCR assay demonstrated excellent analytical accuracy, identifying 100% of the spiked-positive *C. auris* samples from both collection types, underscoring the reliability and robustness of the PCR assay in identifying *C. auris* across varied collection methods. When compared with the traditional culture method, the 3-point ESwab collection method showed 100% agreement, indicating that the PCR assay can serve as a reliable alternative to culture methods, which are often time-consuming and labor-intensive. The PPV and NPV were both 100%, further emphasizing the accuracy and reliability of the PCR assay in identifying true positives and true negatives, with clinical sensitivity and specificity also at 100%, proving the assay’s effectiveness for clinical diagnosis of *C. auris*.

The stability of the PPR mix over a 30-day period was also confirmed. The absence of significant deviations in the CT values indicates that there were no significant fluctuations or degradation in the performance of the PPR mixes over the temperatures and timeframe assessed. The tight standard deviation and CV values further confirm that the assay’s sensitivity and accuracy remained stable throughout the testing period.

The nasal-alone ESwab collection method, compared against the 3-point ESwab method among patients previously diagnosed with *C. auris* colonization, detected *C. auris* in 86.2% of the presumptive positive specimens, showing slightly lower clinical sensitivity than the 3-point collection method. Clinical specificity was 100% for both specimen types. The NPV of 94.3% indicates a high proportion of true negatives among the negative results, supporting the reliability of the nasal-alone method for screening purposes. The overall agreement for the nasal swab method was 96.0%. The high agreement rates and accuracy metrics support the implementation of PCR-based diagnostics as a reliable and efficient approach for detecting *C. auris*, potentially leading to faster diagnosis and improved patient outcomes.

These findings have significant implications for clinical practice, suggesting that as broader screening strategies become necessary, both methods can effectively be utilized depending on the specific needs and constraints of the strategies employed within a healthcare setting. NYS available data have shown that 3-point collection (nares/axilla/groins) provides the greatest yield when screening for *C. auris* (16). This finding is noted in our study in that four samples that had identified *C. auris* on 3-point were not identified on that patient’s nasal swab. However, the swabbing of distinct body sites using one single swab may possibly dilute the concentration of *C. auris* collected or even risk of transferring pathogenic microbes between anatomic sites. Additionally, collection of 3-point swabs can be somewhat challenging for both healthcare providers and patients and may limit broadening screening strategies. Single swabs for one site remain a reliable and efficient approach for patient screening. Recent studies have identified the nares as the most sensitive single site for detecting *C. auris* colonization, harboring higher fungal loads compared with other commonly swabbed sites like the axilla and groin (16–18). Although combining swabs from multiple sites may increase clinical detection of cases, targeting the nasal alone offers a potentially more practical approach for broader patient screening.

Limitations of the current work include the fact that current clinical screening strategies at our institution are limited to patients with invasive ventilatory support. Conclusions regarding simpler collection methods for broadening screening strategies to patients in less-intensive settings may not be directly generalizable. Given the limitations in sample volume and the timing of testing, nasal samples were assessed for clinical accuracy comparing their results with that of patients’ 3-point collection and not culture. It is possible that the performance of nasal swabs with direct comparison to a more appropriate comparator could yield superior performance. Further studies directly comparing the clinical performance between 3-point and nasal ESwab in diverse patient populations and settings would be beneficial. This would help clarify performance, refine screening protocols, and ensure the most effective detection of *C. auris*, ultimately aiding in the control and prevention of healthcare-associated outbreaks.

## Data Availability

Data is available upon request. Please reach out to the corresponding author.

## SUPPLEMENTAL MATERIAL

Table S1 spectrum.02114-24-s0001.pdf Analytical specificity.

ASM does not own the copyrights to Supplemental Material that may be linked to, or accessed through, an article. The authors have granted ASM a non-exclusive, world-wide license to publish the Supplemental Material files. Please contact the corresponding author directly for reuse.
