# Supplementary material for: Performance characteristics of an automated, high-throughput RT-PCR assay for the detection of Candida auris on 3-point and nasal swabs
Source: Microbiol Spectr. 2025 Feb 14;13(4):e02114-24. doi: 10.1128/spectrum.02114-24 (PMC11960114; doi:10.1128/spectrum.02114-24)
Supplement: Table S1 — Analytical specificity. [file spectrum.02114-24-s0001.pdf]

Supplementary Materials:

Table S1. Analytical specificity: Differences in cycle threshold (CT) values for *C. auris* (inoculation of 5.00 Log CFU/mL) and co-colonizing organisms (inoculation of yeasts: 4.32 Log CFU/mL; bacteria: 5.49 Log CFU/mL) in 3-point and nasal-alone ESwab matrices.

**Organism**	**Source**	**Differences in *C. auris* CT value**	
		**Nares/Axilla/Groin**	**Nasal-alone**
Candida duobushaemulonii	CDC AR Bank #0391	0.00	-0.10
	CDC AR Bank #0392	0.30	-0.40
Candida haemulonii	CDC AR Bank #0393	-0.20	-0.10
	CDC AR Bank #0395	0.30	-0.20
Kodameae ohmeri	CDC AR Bank #0396	-0.50	-0.30
Pichia kudriavzevii	CDC AR Bank #0397	-0.70	-0.20
(Candida krusei)	Clinical specimen	0.30	-0.40
Clavispora (Candida) lusitaniae	CDC AR Bank #0398	-0.60	-0.20
	Clinical specimen	0.40	-0.50
Candida albicans	ATCC 14053	-0.60	-0.20
	Clinical specimen	0.10	-0.40
Candida tropicalis	ATCC 950	-0.40	-0.10
	Clinical specimen	0.00	-0.40
Candida parapsilosis	ATCC 22019	-0.60	-0.30
	Clinical specimen	0.10	-0.30
Methicillin-resistant *Staphylococcus aureus (MRSA)*	Clinical specimen	-0.70	-0.40
	ATCC 43300	-0.10	-0.30
Methicillin-susceptible *Staphylococcus aureus (MSSA)*	Clinical specimen	-0.20	-0.30
	ATCC 43300	0.40	-0.30
Staphylococcus epidermidis	Clinical specimen	-0.40	-0.10
	ATCC 29213	0.30	-0.50
Staphylococcus hominis	Clinical specimen	-0.20	-0.10
	ATCC 12228	0.00	-0.50
Escherichia coli	Clinical specimen	-0.40	0.00
	ATCC 13846	-0.30	-0.50
Standard Deviation (SD)		**0.20**	**0.10**
